# Thomas Earl Starzl, MD, PhD (1926–2017): Father of Transplantation

**Published:** 2017-05-01

**Authors:** B. Eghtesad, J. Fung

**Affiliations:** 1 Transplant Center, The Cleveland Clinic, Cleveland, Ohio, USA; 2 University of Chicago, Chicago, USA


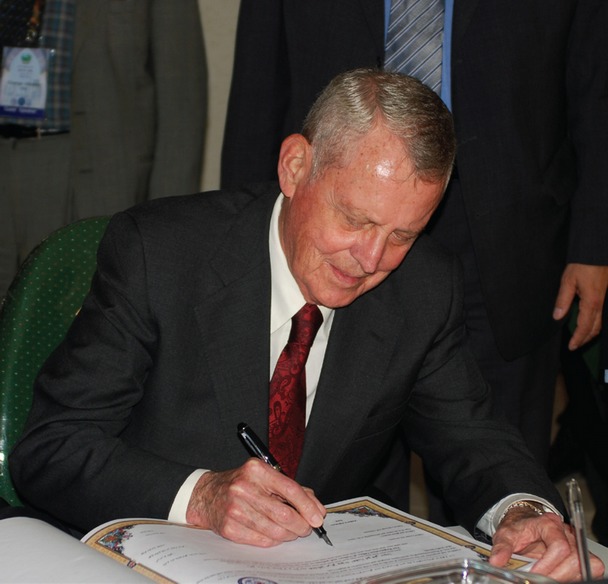


Thomas E. Starzl, MD, PhD, was known to most as the “Father of Transplantation”—he laid the groundwork for an entire new field of medicine. Throughout his career he continued to make among the most significant, landmark advancements, from technically describing and perfecting the many variations of liver surgery, including transplantation, to identifying better ways to control organ rejection, to offering more precise understanding of actual disease processes, including discoveries about organ acceptance, which have completely changed the face and conventional paradigms of transplant immunology.

Although he retired from clinical and surgical service in 1991, Dr. Starzl devoted his time to researching endeavors and remained active as professor of surgery at the University of Pittsburgh School of Medicine and the University of Pittsburgh Medical Center’s (UPMC Health System) program named after him, the “Thomas E. Starzl Transplantation Institute.” He has earned the additional distinctions of being one of the most prolific scientists in the world as well as the most cited scientists in the field of clinical medicine.

Dr. Starzl was born on March 11, 1926 in LeMars, Iowa, the son of a newspaper editor. He attended Westminster College in Fulton, Missouri, where he earned his bachelor’s degree in biology. He went on to the Northwestern University Medical School in Chicago, where in 1950 he received a master’s degree in anatomy and in 1952 earned both a PhD in neurophysiology and an MD with distinction. Dr. Starzl pursued his interest in surgery and research with a residencies and fellowship at Johns Hopkins, the University of Miami, and the Northwestern Universities. He was a Markle Scholar in Medical Science, a distinguished honor bestowed annually to a small group of exceptionally promising young physicians in academic medicine. Dr. Starzl served on the faculty of Northwestern University from 1958 to 1961 and joined the University of Colorado School of Medicine as an associate professor in surgery in 1962. He was promoted to professor in 1964 and appointed chairman of the department of surgery from 1972 to 1980. Dr. Starzl joined the University of Pittsburgh School of Medicine in 1981 as professor of surgery. Until 1991, he served as chief of transplantation services at University of Pittsburgh’s Presbyterian Hospital, Children’s Hospital of Pittsburgh, and the Veterans Administration Hospital in Pittsburgh, overseeing the largest and busiest transplant program in the world. He then assumed the title of Director of the University of Pittsburgh Transplantation Institute, a post which permitted his full attention to research. In 1996, the Institute was renamed in his honor.

Dr. Starzl performed the world’s first successful series of human kidney transplantations starting in 1962 and then with human liver transplantation in 1963, with the first successful liver transplantation in 1967—all at the University of Colorado University and Denver Veterans Administration Hospitals. Among his many enduring accomplishments to the field of transplantation, Dr. Starzl determined that rejection could be controlled with corticosteroids as an addition to azathioprine. This theory proved successful and was subsequently used with anti-lymphocyte globulin, then with cyclosporine, and finally with tacrolimus. It was this development that advanced transplantation from an experimental procedure to an accepted form of treatment for patients with end-stage diseases of the liver, kidney, and heart. It also allowed surgeons to explore the feasibility of transplanting other organs, such as the pancreas and lung. 

Dr. Starzl and his team at the University of Pittsburgh Medical Center had been instrumental in the development of tacrolimus (then known as FK506) since 1986. In 1989, Dr. Starzl announced the first-time clinical use of tacrolimus, presaging yet another significant advancement in transplant medicine, whereby patient and graft survival rates for liver and other organ transplants greatly improved and successful intestine transplantation was for the first time possible—in 1994, the US Food and Drug Administration (FDA) approved the drug for clinical use. He also led the research team that examined the feasibility of xenotransplantation to close the gap on the chronic shortage of human organs. In 1992 and 1993, Dr. Starzl’s team made medical history when surgeons performed two baboon-to-human liver transplants. Dr. Starzl himself had performed six baboon kidney transplants in 1963 and 1964 and the world’s first chimpanzee liver transplants in three children between 1969 and 1974.

Among the over 225 awards and honors bestowed to Dr. Starzl are the Brookdale Award in Medicine presented by the American Medical Association Board of Trustees and the Brookdale Foundation for significant contributions to the field of clinical medicine, teaching and research; the William Beaumont Prize from the American Gastroenterological Association for outstanding contributions to the field and practice of gastroenterology; the Peter Medawar Prize of The Transplant Society; the Jacobson Innovation Award of the American College of Surgeons; the Gustav O. Lienhard Award of the Institute of Medicine of the National Academy of Sciences; and the Lasker Award from the Albert and Mary Lasker Foundation and the Benjamin Franklin Award from the American Philosophical Association. 

Dr. Starzl’s national and international involvement included membership in more than 58 professional and scientific organizations, including election as president of the International Transplantation Society, founding president of the American Society of Transplant Surgeons and founding president of the Transplant Recipients International Organization. In 1992, he was inducted as one of only five American members into the prestigious National French Academy of Medicine. He belonged to the editorial boards of 22 professional publications and authored or co-authored more than 2250 scientific articles, four books and more than 300 book chapters. According to the Clarivate Analytics (formerly Institute for Scientific Information [ISI]), Dr. Starzl once published a paper every 7.3 days, on average, making him one of the most prolific scientists in the world. In 1999, ISI identified Dr. Starzl as the most cited scientist in the field of clinical medicine, a measure of his work’s lasting influence and utility. The book “1000 Years, 1000 People: Ranking the Men and Women Who Shaped the Millennium,” placed Dr. Starzl 213^th^ on its list of those whose contributions have significantly influenced history’s progress.

Soon after arriving Pittsburgh, Dr. Starzl had written that “what was inconceivable yesterday, and barely achievable today, often becomes routine tomorrow.” Liver transplantation proved to be a prime example. Dr. Starzl had begun his experimental liver transplant studies in 1958 with almost no resources at the same time as the tentative first steps were taken to land a man on the moon. The objective of replacing a diseased liver with a healthy one was viewed by critics as a fantasy and the series of early failures appeared to confirm their predictions. Instead of abandoning his efforts, Dr. Starzl called upon an inner strength to not only perfect the technique, but also promote social and medical acceptance of transplantation through tireless efforts to educate scientists, other professionals, and the public. Determined to share any knowledge that might help others, Dr. Starzl has devoted significant energy to rapidly disseminating his discoveries. He has personally trained a large proportion of the surgeons and basic scientists who have set up transplantation programs around the world. As noted by Dr. Ben Eiseman, a colleague of Dr. Starzl while he was at the University of Colorado (Eiseman B. The puzzle people: memoirs of a transplant surgeon. *Arch Surg* 1992;**127**:1009-112):

“He had the ability to attract a diverse group from many walks of life to his surgical team. Even more important, he stimulated his team to superhuman effort by the sheer excitement and sense of importance of the grand experience of which each member was a part. Seldom has any surgeon been able to multiply the effectiveness of his own hands so completely by involving others in his experiments in the laboratory or on the wards as did Starzl. He was always out in front and never asked of anyone else more than he asked of himself.”

On a personal note: “To all those that worked with Dr. Starzl—you all understand his contributions to medicine and science, you know what his vision and insight have provided to literally millions of patients who benefitted from his quest for knowledge. To those that lived with Dr. Starzl—you know that he was brutally honest—both in critique and in praise—you earned whatever opinion he thought of you. To those that loved Dr. Starzl—you know we will miss him terribly but are all better people for having the benefit of knowing him. You know of his love for people and things that he did to help others, but there were things he did to help others that few knew about and for which he refused to take credit for.”

Dr. Starzl is survived by his wife of 36 years, Joy Starzl; a son, Timothy Starzl, and a grandchild, Ravi Starzl, PhD. He was preceded in death by a daughter, Rebecca Starzl, and a son, Thomas F. Starzl.

